# Allied health – 3006. Homeopathy in treating allergic rhinitis - An interventional pilot study

**DOI:** 10.1186/1939-4551-6-S1-P182

**Published:** 2013-04-23

**Authors:** Shubhamoy Ghosh, Subhranil Saha

**Affiliations:** 1 Pathology & Microbiology, Mahesh Bhattacharyya Homoeopathic Medical College and Hospital, India

## Background

This research aims at exploring the efficacy of homeopathic medicines in allergic rhinitis whose prevalence is increasing at an alarming rate throughout the world. India has an estimated number of 15-20 million patients with asthma and 30-80% of these suffer from allergic rhinitis. So, allergic rhinitis is considered as a major chronic respiratory disease due to its prevalence, impact on quality of life, work/school performance and productivity, economic burden and links with asthma.

## Methods

Single arm, experimental, interventional, prospective, non-randomized, before and after comparison pilot study without control was carried on thirty participants suffering from Allergic Rhinitis. The trial was aimed to assess the efficacy of homeopathic remedies, chosen strictly on individualization and symptom similarity, in bringing changes in serum Immunoglobulin E (IgE) level, absolute eosinophil count and allergic rhinitis symptom scores (approved by Institutional Review Board) by comparing the score before medication (baseline) with score after medication.Institutional ethical clearance was obtained; then thirty four consenting patients were enrolled after screening by eligibility criteria and were allocated to classical Homeopathic treatment, out of which four cases were dropped out and thirty cases were regular. Outcome measures were assessed and analyzed after one year.

## Results

After one year of Homeopathic treatment, reduction in serum IgE level (1006.83±395.17 versus 336.5±126.96), absolute eosinophil count (600.33±103.61 versus 302.5±82.21) and symptom score (30.27±5.12 versus 12.83±2.72) were statistically highly significant (paired *t* test; *t*_29 =_ 10.84, 18.17 & 22.37 respectively; P = 0.0000) with 95% confidence level. No adverse effects and/or complications were observed.

## Conclusions

Data suggest that individualized Homeopathic treatment may be a useful measure for the patients suffering from Allergic Rhinitis. However Randomized Controlled Trials (RCTs) with larger sample size and longer duration should be undertaken for confirmation of the conclusion.

